# The impacts of unstructured nature play on health in early childhood development: A systematic review

**DOI:** 10.1371/journal.pone.0229006

**Published:** 2020-02-13

**Authors:** Kylie A. Dankiw, Margarita D. Tsiros, Katherine L. Baldock, Saravana Kumar

**Affiliations:** 1 School of Health Sciences, University of South Australia, Adelaide, Australia; 2 Alliance for Research in Exercise, Nutrition and Activity, School of Health Sciences, University of South Australia, Adelaide, South Australia; 3 Australian Centre for Precision Health, University of South Australia Cancer Research Institute, Adelaide, South Australia; Pontificia Universidad Catolica de Chile, CHILE

## Abstract

**Background:**

Nature play is growing in popularity as children’s play spaces are transforming from traditional playgrounds into more nature-based play spaces with considerable financial and resource investment from government bodies. This has resulted in the re-development of children’s play spaces to incorporate more natural elements such as trees, plants and rocks. Despite this, it is unclear whether there is empirical evidence to support claims that play in nature is beneficial for child health and development.

**Aim:**

To conduct a systematic review examining the impacts of nature play on the health and developmental outcomes of children aged 2–12 years.

**Methods:**

Seven electronic databases were searched (MEDLINE, ERIC, Embase, PsycINFO, The Cochrane Library, The Joanna Briggs Institute and Emcare) from inception to July/August 2018 (search updated July/August 2019). The Inclusion criteria were children aged 2–12 years with no health/developmental conditions. The exposure/intervention of interest was unstructured, free play in nature. Critical appraisal of included studies was conducted using the McMaster Critical Appraisal Tool. Descriptive synthesis was then undertaken using the NHMRC FORM Framework.

**Results:**

Out of 2927 articles identified, 16 studies met the inclusion criteria. The nature play exposure/intervention was heterogeneously described, and a plethora of outcome measures were used. Nature play had consistent positive impacts on physical activity outcomes and cognitive play behaviours (imaginative and dramatic play). However, there remain some concerns regarding the quality of the evidence base, heterogeneity in intervention description and parameters in the outcome measures used.

**Conclusions:**

While the positive impacts of nature play were encouraging in terms of physical activity and cognitive development, nature play stakeholders should focus on producing a universal definition for nature play, the development of standardised outcome measures and the conduct of robust research designs. Implications of these findings suggest the need for the development of standardised guidelines to inform practice and policy in the design of children’s play spaces in different contexts.

## Background

There is growing concern regarding the physical and mental wellbeing of children [1–3]. Research indicates that from 1990 to 2016 the number of children aged between 0–5 years considered overweight or obese increased from 32 million to 41 million globally [2], and as these children get older 10–20% will develop a mental health disorder between the ages of 5–17 years [1]. While the causes of childhood obesity and its impact on physical and mental well-being are complex and multifactorial, evidence consistently highlights the positive effects of outdoor physical activity (PA) in ameliorating these risks in the short and long term [3–5].

In addition to the importance of PA for children’s health and wellbeing, spending time outdoors and engaging in activities such as play has been demonstrated to improve emotional and social resilience in children [6, 7]. Play is an essential part of childhood as it facilitates developmental outcomes, such as gross motor skills, as well as cognitive and social development [8–10]. Despite these benefits, research indicates that access to outdoor play continues to diminish amongst children due to a plethora of reasons such as access, safety, time and competing interests [11–13]. This lack of outdoor play may promote a sedentary lifestyle with play restricted to electronic devices and solitary play [11, 14], suggesting that children may be becoming ‘nature deficient’, failing to develop a personal connection to the natural world [15–17].

Several initiatives have been trialled as means of improving children’s access to and engagement with outdoor play with varying degrees of success [18]. One recent initiative is nature play [3, 19, 20], a term developed by categorising children’s play into a more specific type/form of play which is unstructured and nature-based [19]. Nature play environments can be characterised as having natural elements such as plants, rocks, mud, sand, gardens, forests and ponds or water [18, 21].

Emerging literature has explored the health outcomes of children engaging in nature play. For instance, some studies have found positive outcomes in children’s mental health [13], PA [22], academic performance [23], social development [3, 24, 25] and cognitive development. Research is therefore suggesting that children may be more physically active, social and psychologically resilient when engaging in nature play activities.

A systematic review by Mygind et al [26] of 84 studies, found similar outcomes for children and adolescents, relating to mental health improvements after an immersive nature experience. However, their review focused on children and adolescents with behavioural and/or emotional disturbances and employed a broader definition (linked to nature experience, rather than play). The scope of the review was also limited to studies published from 2004–2017. Similarly, another systematic review [27] of 61 primary research studies also highlighted numerous physical and mental health benefits for children engaging with nature, although there were many methodological concerns with the review. The methods used to conduct this review were inconsistent with accepted systematic review methodologies and reporting [28]. Specifically, the review [27] was not registered with PROSPERO [29]; it lacked a comprehensive search strategy (no database search), and did not demonstrate rigour in synthesising the results (no second reviewer) [28]. Furthermore, the review is now a decade old, and to our knowledge, no other systematic reviews have investigated the impacts of children’s engagement with unstructured nature play. It is therefore timely to update the evidence base on the impacts of nature play on children’s health and development using rigorous methodologies. The purpose of this systematic review was to identify the impact of participating in nature play on health and developmental outcomes in children aged 2–12 years.

## Methods

### Protocol and registration

An a priori protocol was developed and registered with PROSPERO (registration number: CRD42018084764). This review was informed by, and reported using, the Preferred Reporting Items for Systematic Reviews and Meta-Analyses (PRISMA) guidelines [28] (S1 Appendix).

### Search strategy

The following electronic databases were searched: MEDLINE, ERIC, Embase, PsycINFO, The Cochrane Library, The Joanna Briggs Institute and Emcare. All databases were searched from inception to July/August 2018 (search updated July/August 2019). Grey literature searching was conducted through Pro Quest thesis dissertations, Mednar, Children & Nature Network Research Library, Nature Play website (SA, WA, ACT, QLD) and the South Australian Department for Education and Development websites. The references of relevant articles were also searched to identify potential additional articles (pearling). Google and Google Scholar were also searched using the terms ‘nature play and children’ in the advanced search option, studying the first 100 results [30]. The following search terms were used with relevant Boolean Operators and MeSH terms identified for individual databases: child, children, pre?school, primary school?, elementary school?, kindergarten? or early?child* or early learning or early?learning or boy? or girl? or toddler?, garden? or forest? or grassland? or wetland? or wilderness or nature* or natural or out?door? or out?side? or park?) adj3 (free?play* or play or plays or playing or played or recreat* or unstructured activit* green school? or green?space? or forest school? or out?door? education or environmental play or "play* in the environment"). The search was limited to humans and English language.

### Study designs

All quantitative primary research studies were considered, excluding studies with a qualitative research paradigm. Qualitative research was excluded in this review as the review question was aimed at identifying the empirical evidence to determine the impacts of nature play on child health/development. The focus of the review was not to explore and understand perspectives about and experiences of nature play. The inclusion and exclusion criteria for the population-exposure-outcome (PEO) are outlined below.

### Population

The included participant population was children aged 2–12 years with no pre-existing/diagnosed physical, mental, behavioural or neurological health conditions.

### Exposure

Studies were included if the exposure or intervention involved unstructured, free play within nature (forest, green spaces, outdoors, gardens) and included natural elements (highly vegetated, rocks, mud, sand, gardens, forests and ponds or water). Studies were excluded if the exposure or intervention involved structured activities such as sport and other organised activities (such as but not limited to outdoor education programs with structured activities, orienteering). The rationale for excluding outdoor education programs was decided upon after an extensive review of the literature (undertaken prior to the conduct of this systematic review), which highlighted that educational programmes commonly follow structured activities in nature and follow a curriculum. The literature shows that educational programmes are commonly led by educators (structured) which is inherently different from how this review described nature play, as unstructured and child-led play, free play [31, 32]. Similarly, studies were also excluded if the exposure or intervention involved an artificial environment (playgrounds with slides, swings etc) unless it was used as a comparator.

### Outcome

Studies with outcomes that were measured by proxy or objectively were included. Studies measuring at least one relationship/outcome from exposure to nature play were included, such as but not limited to, gross motor, social, cognitive development, learning, quality of life, emotional health, mental health, physical health (weight, body mass index), PA and moderate to vigorous physical activity (MVPA).

### Study selection

Literature selection was underpinned by a three-stage process. Stage 1 included the input of all studies searched from each database into EndNote X9 ^®^ (2019, Clarivate Analytics, Toronto, Canada) [33], where duplicates were removed. Stage 2 involved exporting the studies from EndNote X9 ^®^ into the online Covidence^TM^ software (2019, Alfred Hospital in Melbourne Australia, Instituto de Efectividad Clinica Y Sanitaria (EROS) in Buenos Aires, Argentina). The screening of title and abstracts was conducted in duplicate by four independent reviewers (KD, MT, KB, SK) using Covidence^TM^. One reviewer screened all studies and the remaining three reviewers divided all studies evenly amongst themselves. Eligibility was based on the inclusion and exclusion criteria. Any disagreements were discussed by the review team until consensus was achieved. Stage 3 involved assessing the eligibility of article full text according to inclusion and exclusion criteria. Reasons for exclusion of studies were recorded. Conflicts were resolved through discussion with the review authors.

### Methodological quality

The McMaster Critical Appraisal Tool (CAT) for Quantitative Studies was used to assess the quality of the included studies [34]. The McMaster CAT was used as it is an appropriate generic tool for all quantitative research designs. While there are many CATs’, there is no gold standard CAT [35] and the McMaster CAT has been used widely used by other systematic reviews [36, 37]. The McMaster CAT covers eight main components: study purpose; literature review; study design; sample (participants described, justification of sample size); outcomes (reliability, validity of outcome measures and outcomes); intervention (intervention described in detail, contamination and co-intervention avoided); results (statistical and clinical significance, analysis procedure and dropouts); conclusions and implications for practice (limitations and bias). For the purpose of this review, the McMaster CAT for Quantitative Studies was modified due to the study designs. For example, questions about randomisation were removed due to the lack of randomised controlled trial (RCT) study designs. Similarly, questions about contamination and co-intervention were also not applicable in most cases because studies only included one group or two groups in different locations. The individual components of the tool were rated as ‘yes’ (would receive a 1), ‘no’ (would receive a 0), ‘not addressed (NA)’ (would receive a 0) and if ‘not applicable’ was applied this was taken off the total score, which could be a maximum of 14. The methodological quality of the included studies was assessed by the same four independent reviewers, whereby one reviewer assessed all studies and the remaining three reviewers divided the included studies evenly amongst themselves resulting in each study being assessed twice. Any disagreements were discussed by the review team until consensus was achieved. Studies were classified according to study design using the NHMRC levels of evidence [38].

### Data management

Data extraction from all included studies was conducted by KD using a pre-determined data extraction proforma in Microsoft Excel (version 1811, © Microsoft Cooperation 2018). Duplicate data extraction was conducted for 25% of the included studies (n = 16) and evenly distributed between SK, KB, MT (n = 4) with no discrepancies in extracted data identified. Extracted data included; participant characteristics, sample size, exposure/intervention, comparators/controls, and outcomes (Table 1). Some additional data extracted included, outcome domains (PA, cognitive, emotional, social) outcomes measures, outcome results (statistical significance and descriptive and data) where reported. A meta-analysis of included studies was not feasible due heterogeneity in methodological approaches and a lack of consistency in outcome variables. Therefore, a descriptive synthesis of the results was undertaken.

**Table 1:** Level of evidence and modified Mc Master results of methodological quality.

Study	NHMRC level and study design	Modified McMaster tool items														Raw score %
		1	2	3a	3b	4a	4b	5a	5b	5c	6a	6b	6c	6d	7	
Brussoni et al [75]	Case series pre-test/post-test (mixed methods) level IV	Y	Y	Y	N	Y	Y	Y		Y	Y	Y	Y	Y	Y	12/13 92
Dowdell et al [20]	Case series post-test (mixed methods) Level IV	Y	Y	N	N	N	NA	Y	Y	NA	N	N	N	NA	N	4/14 28
Drown [86]	Interrupted time series Level III-3	Y	Y	Y	N	Y	N	Y			Y	Y	Y	N	Y	9/12 75
Fjortoft [76]	Non-randomised experimental Level III-2	Y	Y	Y	N	Y	Y	Y	NA	Y	Y	Y	Y	Y	Y	12/14 85
Fjortoft 2001 [82]	Non-randomised experimental Level III-2	Y	Y	Y	N	Y	Y	Y	NA	Y	Y	Y	Y	Y	Y	12/14 85
Gardner and Kuzich [77]	Non-randomised experimental Level III-2	Y	Y	Y	N	NA	NA	Y	N	N	N	Y	Y	NA	Y	7/14 50
Groves and McNish [85]	Case series pre-test/post-test (mixed methods) Level IV	Y	Y	N	N	NA	NA	Y	NA	NA	Y	Y	Y	NA	N	6/14 42
Kuh [83]	A comparative study without concurrent controls (mixed methods) level III-3	Y	Y	N	N	Y	NA	Y	NA	NA	Y	N	Y	Y	Y	8/14 57
Larson et al. [78]	Case series pre-test/post-test Level IV	Y	Y	Y	N	Y	NA	Y			N	N	Y	NA	Y	7/12 58
Luchs and Fikus [74]	Case series pre-test/post-test Level IV	Y	Y	Y	Y	Y	Y	Y			Y	Y	Y	Y	Y	12/12 100
Schweighardt [73]	Case series post-test Level IV	Y	Y	Y	Y	Y	Y	Y			N	Y	Y	NA	Y	10/12 83
Storli and Hagen [81]	Case series post-test Level IV	N	Y	N	Y	Y	Y	Y			Y	Y	N	Y	Y	9/12 75
Torkar and Rejc [5]	Case series post-test Level IV	Y	Y	N	N	NA	NA	Y			Y	Y	Y	Y	Y	8/12 66
Wojciehowski and Ernst [84]	Comparative study (with concurrent controls) Level III-2	Y	Y	Y	N	N	Y	Y	NA	Y	Y	Y	Y	Y	Y	11/14 78
Zamani [80]	Case series post-test (mixed methods) Level IV	Y	Y	Y	N	Y	NA	Y			Y	Y	Y	NA	Y	9/12 75
Zamani and Moore [87]	Case series post-test Level IV	Y	Y	N	N	N	N	Y			Y	Y	Y	N	Y	7/12 58

**KEY: McMaster items to be scored:** 1. Was the study purpose clearly stated? 2. Was the relevant background literature reviewed? 3.Describe the justification of the need for this study? 3a. The sample described in detail? 3b. Was the sample size justified? 4a. Were outcome measures reliable? 4b. Were the outcome measures valid? 5a. Was the intervention/exposure described in detail? 5b. Was contamination was avoided? 5c. Co-intervention was avoided? 6a. Results were reported in terms of statistical significance? 6b. The appropriate analysis used? 6c. Clinical importance reported? 6d. Dropouts recorded? 7. Conclusions were appropriate given the study methods and results? Y = yes, N = no, NA = not addressed and column coloured out in grey = not applicable.

### Synthesis of results

The data extraction process highlighted that the included studies used numerous outcome measures. While some studies used comparable instruments to measure outcomes, the constructs and data analysis methods were highly heterogeneous, thereby precluding a meta-analysis (see results section). Therefore, descriptive synthesis was undertaken. The descriptive synthesis was conducted using the NHMRC FORM framework [39]. This widely adopted framework [37] was used to facilitate a consistent and transparent approach to data synthesis, a common limitation of many public health systematic reviews [40]. The FORM framework focuses on the descriptive information at the evidence-base of included studies across five main components to formulate and grade recommendations for practice guidelines [39]. The grades range from A; excellent, B; good, C; satisfactory and D; poor (see Table 4). The five components are represented by 1) evidence base (NHMRC hierarchy of evidence, risk of bias), 2) consistency (across included studies), 3) clinical impact (measure of the likely benefit across the target population), 4) generalisability (clinical question answered) and 5) applicability (setting, organisational and cultural factors in an Australian context) [38]. The fifth component, ‘‘applicability” was not considered during the data synthesis process for this systematic review due to its international focus. Once a rating for each of the components was undertaken based upon all included studies, an overall recommendation from the ratings for each individual component was determined. Each component of the FORM framework was discussed during a panel meeting involving all four reviewers, where grades for each component and an overall recommendation was agreed upon by consensus. The grades of the recommendations described the strength of the body of evidence underpinning the recommendation.

## Results

### Study selection

**Fig 1:**
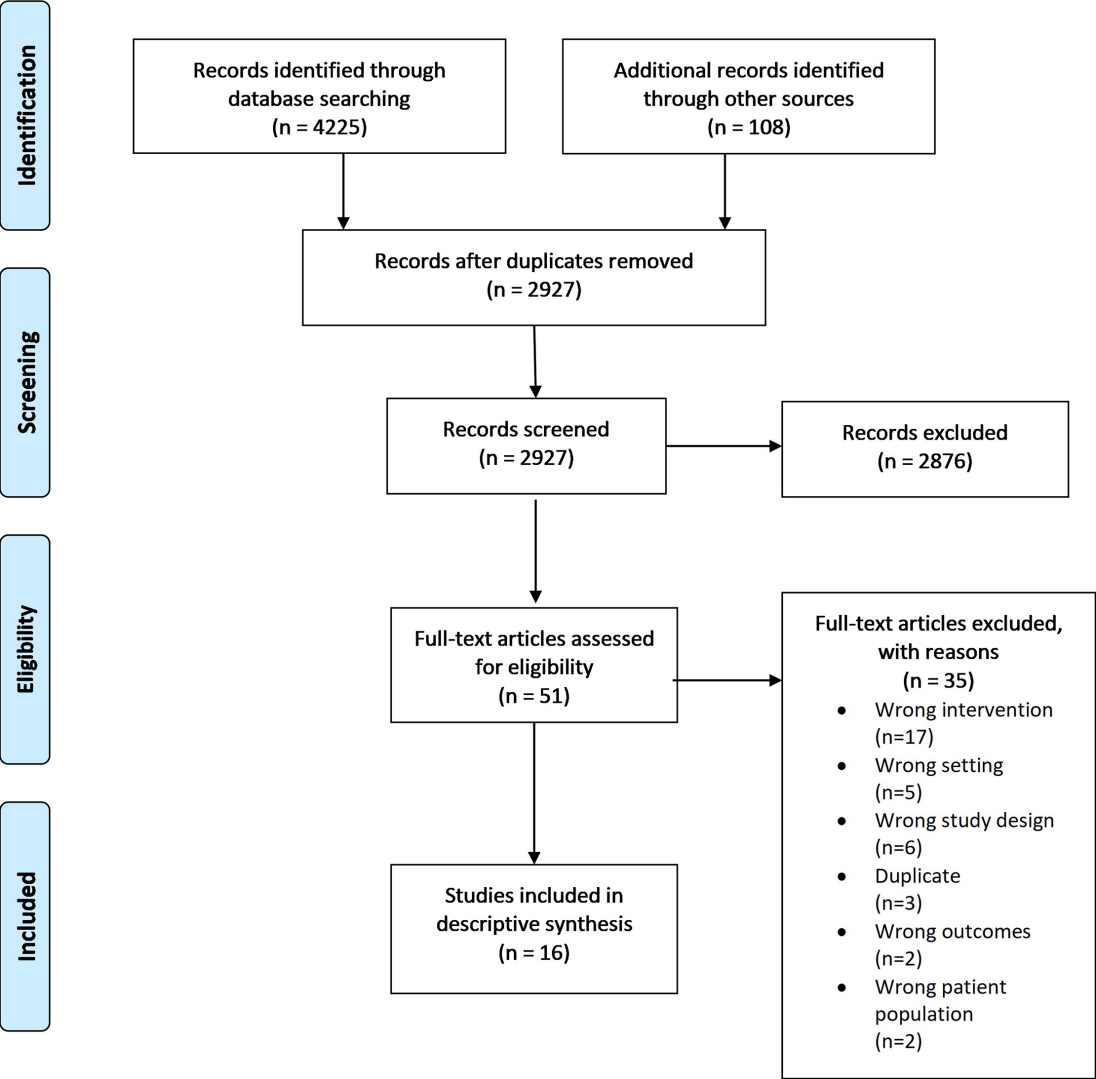
PRISMA flowchart.

Searching identified 4225 studies from databases and other sources (Fig 1). After removal of duplicates (n = 2927) and screening of records, 16 studies were deemed eligible for inclusion in the review. A total of 35 studies were excluded; 17 for ineligible intervention [41–57], 5 for ineligible setting [58–62], 6 for ineligible study design [63–68], 2 for ineligible outcomes [69, 70], 2 for ineligible patient population [71, 72] and 3 additional duplicates were detected and excluded [73, 74].

### Risk of bias within studies

The critical appraisal scores for each of the included studies and the NHMRC level of evidence is described in Table 1. There were several methodological concerns amongst the included studies. The main concerns were: sample size not being described in detail (only eight studies described their sample [73–80]; the lack of sample size justification (only three studies did this [73, 74, 81]; lack of reliability and validity of outcome measures [ten studies reported on reliability [73–76, 78, 79, 81–84] and seven reported on validity [73–76, 81, 82, 84]; and finally the lack of reporting dropouts, with only six studies reporting dropout rates [5, 74, 75, 81, 83, 84].

### Study characteristics

The study characteristics from the 16 included studies are displayed in Table 2. A range of study designs were identified, as were the geographical study locations, which included Norway [76, 81, 82], Slovenia [5], Australia [20, 77], United Kingdom [77], Scotland [85], Canada [75], United States of America [73, 78, 83, 84] and Germany. The studies were conducted from 2001 onwards to the most recent three studies published in 2018 [74, 77, 84] which may indicate the relatively recent interest in nature play.

**Table 2:** Study characteristics.

Study and year	N	Age range (years)	Intervention/exposure	Comparator/control	Outcome domains	Method of measurement
Brussoni et al 2017 [75]	45	2–5 years	Nature play space	Traditional play space	PA, emotional, social, play behaviour	Accelerometer, play observations, SDQ, PSBS
Dowdell et al 2011 [20]	12	2–6 years	Nature play space	Traditional play space	Play behaviour	Play observations
Drown 2014 [86]	25	3–5 years	Nature play space	Traditional play space	Play behaviour, social	Play observations, direct observations
Fjortoft 2004 [76]	75	5–7 years	Nature play space	Traditional play space	Motor fitness, functional play, constructive play, symbolic play	Play observations, motor fitness test, EUROFIT
Fjortoft 2001 [82]	75	5–7 years	Nature play space	Traditional play space	Motor fitness	Motor fitness test, EUROFIT
Gardner and Kuzich 2018 [77]	97	8–9 years	Nature play space	Inside classroom using photographs of a forest/bush environment	Learning	Words per poem, content analysis
Groves and McNish 2011 [85]	25	5–6 years	Nature play space	Traditional play space	PA, dramatic play, social play, behaviour change	Pedometer, play observations
Kuh 2013 [83]	90	4–8 years	Nature play space	Traditional play space	Constructive play	Play observations
Larson et al 2014 [78]	8	3–4 years	Outdoor toys. Fixed equipment and open space.	Controlled naturalistic environment	MVPA, social	Direct (OSRAC-P) observations, play observations
Luchs, and Fikus 2016 [74]	17	5–6 years	Nature play space	Traditional play space	PA	Pedometer
Schweighardt et al 2015 [73]	17	3–5 years	Nature play space	Traditional, garden, adventure play space	PA	Accelerometer, direct observation (SOPARC)
Storli 2010 [81]	16	3–5 years	Nature play space	Traditional play space	MVPA	Accelerometer
Torkar and Rejc 2017 [5]	25	4–5 years	Nature play space	Traditional play space	PA	Direct observation, GPS
Wojciehowski and Ernst 2018 [84]	86	3–6 years	Nature play space	Traditional play space	Creativity	Thinking test (TCAM)
Zamani 2013 [80]	36	4–5 years	Nature play space	Traditional play space, mixed play space	Cognitive play, functional play, constructive play, exploratory play, dramatic play	Play observations
Zamani and Moore 2013 [87]	62	4–5 years	Nature play space	Traditional play space	Cognitive play, functional play, constructive play, exploratory play, dramatic play	Play observations

KEY: Nature play space = unstructured, free play within an outdoor environment consisting of natural elements; trees, vegetation, water, sand and trees, Traditional play space = structured activity, play within a man-made play-ground setting, SDQ = Strengths and Difficulties Questionnaire, PSBS = Preschool Social Behaviour Scale, EUROFIT = European Test of Physical Fitness, OSRAC-P = Observational System for Recording Physical Activity in Children–Preschool Version, SOPARC = System for Observing Play and Recreation in Communities, TCAM = Thinking Creatively in Action and Movement

### Participant characteristics

In regard to the participant characteristics, there was a great deal of diversity in terms of sample size and age. While the overall sample size for all included studies equated to 711, the smallest sample size was just 8 [78], compared to the largest sample size of 97 [77]. The age of participants ranged from 2–9 years and most studies examined 2–5 year old’s, compared with only two studies focussing on older children (aged 8–9 years) [77, 83]. Given most studies were conducted in western countries, the reported ethnicity of participants was primarily Caucasian however, most lacked a detailed description of the sample characteristics.

### Exposure/intervention characteristics

The exposure of interest in the current review was nature play, of which there is no universally accepted definition, and this was reflected among the included studies. There was, however, some commonality to describe the exposure/intervention of nature play. For instance, ‘‘natural playscape” [n = 3 studies [76, 82, 83]], ‘‘nature playground” [n = 2, [74, 81]], and ‘‘natural play” [n = 2, [73, 85]]. Remaining studies used varying terminology including ‘‘free play in nature” [77], ‘‘nature-based” [20], ‘‘open space” [78], ‘‘forest (natural) playground” [5], ‘‘nature preschool” [84] and lastly ‘‘natural zone” [80, 87]. Despite variations in terminology used to describe nature play, some consistent characteristics were observed; specifically, free play and interacting with natural elements such as trees, sand, water and vegetation.

## Outcome measures

A range of outcome measures were used to determine the impacts of nature play on different domains of children’s health and development (Table 3). These included: objective assessments of PA [of which two studies using pedometers, [74, 85] and three studies using accelerometers [73, 75, 81]], while two studies used assessments of motor skills and/or health-related fitness [76, 82]. In addition, many studies used observational measures (n = 11, see Table 1) to report on children’s play behaviours, although only two studies (by one author) used the same tool to observe play behaviour. Only one study used a thinking test called the Thinking Creatively in Action and Movement (TCAM) to measure creativity [84]. Five studies used multiple outcome measures within their studies [75, 76, 78, 83, 85].

**Table 3:** Summary of results.

Study	Physical activity	Health-related fitness	Motor skill	Cognitive development									Social	Emotional
		Flexibility	Balance	Coordination	Functional	Constructive	Exploratory	Dramatic	Imaginative	Symbolic	Creativity	Learning		
Brussoni et al	↓*												↑*	+↓*
Fjortoft		↔	↑*	↑*	↔					↔				
Fjortoft		↔	↑*	↑*										
Dowdell et al									↑?				↔?	
Drown								↑*						
Gardner and Kuzich												↑?		
Groves and McNish	↑*												↑?	↑?
Kuh						↑*							↑?	
Larson et al.	↔?													
Luchs and Fikus	↔													
Storli and Hagen	↔													
Schweighardt	↔?													
Torkar and Aljoša	↔													
Wojciehowski and Ernst											↑*			
Zamani						↑?	↑?	↑?	↑?					
Zamani et al						↓?	↑?	↔?	↔?	↑?				

KEY: ↑ = increase, ↓ = decrease, ↔ = no change, (+) = positive change/improvement, (-) = negative change/improvement

* = statistical significance (p<0.05), (?) = significance not reported or tested, blank cells indicate outcome was not measured in the given study

### Physical activity

Seven studies reported on PA outcomes [5, 73–75, 78, 81, 85]. The two studies using pedometers [74, 85], derived different variables for PA outcomes (gait cycles and steps), and the three studies using accelerometers [73, 75, 81] used different variables to report PA (pecentage of time, time in minutes, step counts per minute). Despite two studies using acceleometry and three studies using acceleometry, different data reduction methods were used and the resultant outcomes of interest were different, meaning the data could not be combined. One study reported a statistically significant increase (p<0.05) in PA using pedometers (steps) from 3,836 steps before a exposure/intervention of nature play compared to 5,104 steps after an exposure/intervention of nature play [85]. Another study [75] reported a statistically significant decrease of 1.32 minutes in MVPA during a 20-minute play period following the nature play re-development of traditional play spaces (p<0.001) Three studies reported no significant change in PA from a nature play intervention/exposure relative to traditional play settings using pedometers (p = 0.10) [74], accelerometers (p = 0.629) [88] and GPS (p = 0.132) [5]. The remaining two studies used descriptive statistics, not testing for statistical significance and found no change in PA after nature play exposure (compared with traditional play spaces) when measured using accelerometry derived MVPA and/or direct observations [73, 78].

### Motor development

#### Health-related fitness and motor skills

Two studies reported on health-related fitness (flexibility) and motor skill (balance and coordination) outcomes. Of these studies, both were conducted by the same author and measured PA using some components of the EUROFIT test (Europeans test of physical fitness and motor fitness test) along with 2 additional fitness-related outcome measures [beam walking (balance) and Indian skip test (coordination) [76, 82]]. Given that the same data set was used to report findings in two different publications, it was not appropriate to combine the data. The intervention group (nature play) significantly improved in all components of the EUROFIT test from pre-test to post-test (p<0.05), however in comparison to the control (traditional play space) the intervention group did not significantly improve in flexibility (sit and reach), yet did significantly improve (p<0.001) post-test comparable to the control in balance (flamingo balance test) and coordination (Indian skip test).

### Cognitive development

#### Play

Five studies reported outcomes for cognitive development [20, 76, 80, 83, 87] in categories of functional, constructive, exploratory, dramatic and imaginative play. Three studies [20, 80, 87] used behaviour mapping outcome measures (different from one another), while one used an Outdoor Play Inventory Observation Protocol [83] and the remaining study used play observations made by teachers [76]. Dowdell et al [20] reported an 8% increase in associative and a 4% increase in imaginative play after an exposure/intervention of nature play compared to a traditional play space (variance not reported). Zamani and Moore [87] reported a 40% increase in functional play after an exposure/intervention of nature play compared to a traditional play space. While Zamani [80] found a 2.3% increase in exploratory play, a 7.7% increase in dramatic play and a 18% increase in constructive play after an exposure/intervention of nature play compared to a traditional play space. Fjortoft 2004 [76] found an increase in affordances for constructive play, however did not report descriptive data (means, median, standard deviation). While Kuh, [83] reported a statistically significant increase of constructive play from 3.78 minutes to 9.9 minutes after an exposure/intervention of nature play (p<0.05).

#### Learning

Two studies measured learning outcomes; one measured poetic writing in terms of word count, imagery and figurative language (use of metaphors and similes), against a measure of 12 categories developed through content analysis [77]. The second study measured learning through a range of categories (attainment levels, incidents of difficult behaviour, punctuality, concentration in class, settling time, concentration after play), by using a five-point ranking scale (1 representing poor/low and 5 excellent) [85]. However, both studies did not report results in terms of statistical significance. Though, of the two studies, one reported learning gains in terms of increasing the use of figurative language from 31% to 69% after an exposure/intervention of nature play [77]. The remaining study (85) reported an increase in attention levels, punctuality, concentration in class, settling time, concentration after play and decrease in difficult behaviour after an exposure/intervention of nature play, however descriptive data (mean, media, standard deviation) was not reported [85].

#### Creativity

Only one study [84] measured creativity outcomes in terms of fluency, originality and imagination against an activity protocol. The findings from this research indicated a significant increase (p<0.05) in fluency, originality and imagination for the intervention group (nature play) post-test, compared to the control (traditional play space) post-test (P<0.001).

### Social

Social outcomes were measured by four studies [20, 75, 83, 85]. Four studies reported outcomes of associative, solitary, cooperative and parallel play [20, 75, 78, 83] measured using observational coding protocols. Two studies measured teacher and peer interactions, antisocial behaviour (bullying) and prosocial behaviour, both measured by teachers using a rating scale [75, 85]. While three studies reported improvements in social outcomes [75, 83, 85], Dowdell et al found no difference between a nature play space and traditional play space [20]. Kuh [83] reported a 3.34 minute increase in cooperative play from baseline after an exposure/intervention of nature play, compared to a 4.68 minute decrease in cooperative play from baseline in a traditional play space. Whilst Groves, [85] reported improvements in teacher and peer interaction and prosocial behaviour after an exposure/intervention of nature play, descriptive data (mean, median, standard deviation) were not reported. Brussoni et al [75] reported a significant increase in prosocial behaviour after an exposure/intervention of nature play (P<0.05, CI 1.17–6.91).

### Emotional

Emotional outcomes were reported by two studies [75, 85]. One study [75] measured aggression and ‘depression’ using a PSBS scale (Preschool Social Behaviour Scale), whilst a second study [85] reported an outcome for mood using a five-point ranking scale (1 representing poor/low and 5 excellent). Brussoni et al [75] found a significant decrease in depression and aggression post nature play exposure/intervention (p<0.03), while Groves [85] found a positive increase in mood post nature play exposure/intervention, however descriptive data (mean, median, standard deviation) were not reported.

## Summary of results

**Fig 2:**
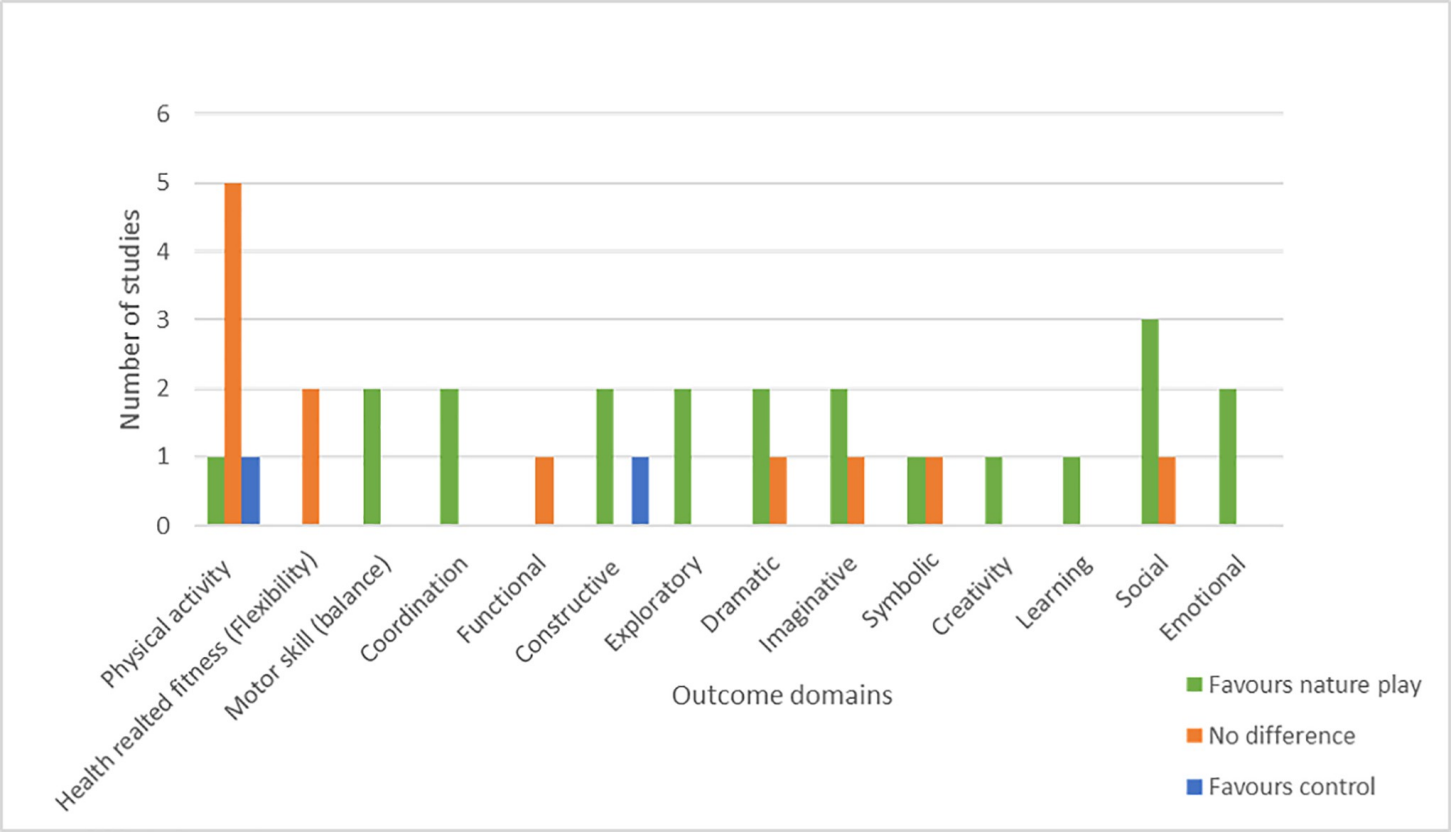
Overview of outcomes and results across studies.

An overview of results across studies is provided by Fig 2 and a summary of the results is provided in Table 3, which highlights the variability of outcomes measured in the included studies (7 broad categories with a total of 12 outcomes). Despite variability in outcome measures, the synthesis of the findings suggests that nature play may have a positive impact on a range of children’s health and developmental outcomes—specifically, PA, health-related fitness, motor skill, cognitive learning, social and emotional development. Regarding PA, after exposure to nature play, one [85] of the seven studies found a significant increase in PA, while one study reported a ~1 minute decrease in PA [75]. Notably, a further five [5, 73, 74, 78, 88] studies reported similar gains in PA when comparing nature play with traditional play space experiences. In terms of cognitive outcomes (play, learning, creativity) [20, 76, 77, 80, 83–85, 87], consistent positive improvements were reported. Such improvements pertained to; poetic writing, attention levels, punctuality, concentration in class, settling time, concentration after play, constructive play, associative play, imaginative play and functional play, however, statistical significance was not reported.

## NHMRC FORM framework

The NHMRC FORM framework was used in the synthesis process, the findings are described in Table 4. However, the results should be interpreted with caution as several methodological concerns were noted, lowering the grade of the evidence base.

**Table 4:** NHMRC form framework analysis.

Component	Grade	Comments
**Evidence base**	C–Satisfactory One- or two-Level III studies with a low risk of bias or Level I or II studies with a moderate risk of bias	Quantity: a total of 16 studies Participants: 711 children aged between 2–12 years Risk of bias: McMaster Critical Appraisal score: low (above 85%), moderate (between 50–80%) and high (Below 50%) Level 111: 6 studies (3 studies with low risk of bias, 2 studies with a moderate risk of bias and 1 study with a high risk of bias) Level IV: 10 studies (3 studies with low risk of bias, 5 studies with a moderate risk of bias and 2 study with a high risk of bias) Overall: 5 studies with a low risk of bias, 7 studies have a moderate risk of bias and 3 studies have a high risk of bias
**Consistency**	C–Satisfactory Some inconsistency, reflecting genuine uncertainty around the question	Inconsistent reporting of statistical significance (only 5 studies reported statistical significance) Consistent descriptive findings of positive outcomes Multiple study designs Varied outcome measures and outcomes Variable exposure/interventions
**Clinical impact**	C–Moderate	No adverse effects reported Risk of harm is low Most studies showed improvements or similar gains (only one study showed a small reduction in PA)
**Generalisability**	B–Good Evidence directly generalisable to target population with some caveats	The population studied across the included studies is accurate to the target population of the systematic review (age range of included studies between 2–9 years) Most studies did not describe their sample in detail, in terms of detailing if the population had no pre-existing/diagnosed health or developmental conditions Studies conducted in eight different countries, with different environment contexts
**Grade of recommendation**	C–Satisfactory Body of evidence provides some support for recommendation but care should be taken in its application	Overall, most studies were found to be low-moderate in terms of methodological quality. While there were consistencies amongst the evidence base in terms of population, geographical location and findings, there was limited clarity and uniformity in outcome measures, outcome domains and exposure/intervention contexts

## Discussion

The purpose of this review was to synthesise the current evidence base on the impacts of nature play exposures/interventions on children’s health and development. The database search yielded 16 eligible studies, with varied research designs. The summary of findings from the included studies highlight that nature play may have positive impacts on a range of health and developmental outcomes for children such as PA, motor development (health-related fitness and motor skill), cognitive development (play, learning and creativity), social and emotional outcomes. However, despite these reported benefits, the current body of evidence has many methodological concerns relating to sample bias, reporting of results and the reliability and validity of outcome measures. Therefore, due to these methodological concerns of the evidence base, the findings of this review and recommendations should be interpreted with caution. To our knowledge, this is the first rigorously conducted systematic review that comprehensively and transparently adds to the current evidence base for nature play. Rigour was achieved by following the PRISMA guidelines, registering the review protocol with PROSPERO, using a comprehensive search strategy including grey literature searching, duplicate screening and assessment of study quality and adopting a widely used framework (NHMRC FORM) to synthesise the strength of the recommendations.

### Physical activity outcomes

The majority of studies which reported outcomes for PA found consistent results to suggest that nature play may positively impact upon children’s PA. The importance of PA to reduce obesity risks has been well documented in the literature [89, 90], particularly in childhood where PA patterns in the early years have been known to track strongly into PA patterns later in life [4]. Similarly, research has consistently shown that children are more physically active when outdoors [4]. Notably, the findings of this review are consistent with previous research which suggests that outdoor play positively influences PA in children [4, 91, 92]. However, what may be an important difference influencing PA, is what specific activities and behaviours take place outdoors, whether they are structured (sports, games) or unstructured (free play) and in what outdoor or environmental context. For example, Barton et al [24] found that children who engaged in unstructured outdoor playground activities compared with structured outdoor orienteering, had higher levels of PA [7]. The findings of this review in conjunction with the extant literature highlights that being outdoors is an important factor in increasing PA for children. Distinctively this review found that nature play spaces are comparable to traditional play spaces in terms of increasing PA. Even though one study (70) reported significantly lower PA during a 20-minute outdoor play period following nature play re-development of traditional play spaces, it is questionable whether a ~1-minute reduction is clinically meaningful. While nature play spaces may not be better than traditional play spaces, there were still positive impacts on children’s PA outcomes. Notably, what may be considered important factors for impacting children’s PA is what behaviours are taking place outdoors and the physical elements of the play space.

### Developmental outcomes

It was consistently highlighted that nature play had positive impacts on developmental outcomes for children, particularly in the cognitive domains of imagination, creativity and dramatic play (n = 5 studies). Imagination, creativity and dramatic play are important aspects of child development as they help children develop a sense of the world around them by allowing them to develop complex thinking skills, emotional intelligence and social skills [93]. A meta-analysis conducted by Fisher [94] emphasised the impact of imaginative play on the development of complex thinking skills for children [94]. Similarly, Singer (1994) found that children aged 3–4 years engaging in pretend play, were observed to be smiling and laughing more compared to children observed in highly structured play environments (teacher centred activities) over a one year period [95]. In addition, imaginative play has also been linked to increased social interaction -this was emphasised by early research in 4-to-5-year-olds showing [96] more positive social outcomes during pretend play compared with structured non-pretend play (i.e. putting a puzzle together).

### Outcomes measures

It was evident throughout the included studies that there was high heterogeneity between outcome measures–this was particularly notable for observational behaviour coding measures. Only two studies from one author [80, 87] used the same outcome measure to code children’s play behaviour. The remaining studies all used varied observational outcome measures, with few reporting reliability data and none reporting information regarding validity. Such variation highlights the lack of a gold-standard tool to observe and code children’s play behaviour–a limitation that made it difficult to establish the consistency of findings between the studies.

### Nature play exposure/intervention

The ambiguity surrounding what constitutes a nature play exposure/intervention was evident throughout the included studies, whereby different terms were used to describe a natural play environment. This diversity in terminology may in part reflect the many different elements and features that may be combined to represent nature play. Furthermore, given that these studies were conducted across a range of geographical contexts, this may have resulted in varied and bespoke nature play environments, developed to suit local needs and requirements. This made it difficult to compare different nature play environments between studies due to the heterogeneity in how the nature play exposure/intervention was described. Similarly, in order to make direct links about the impact of nature play on children’s health and development the exposure/intervention control and comparator play spaces needed to be distinct from one another. However, this was not evident as most studies used a comparator and control environment which were both outdoors. The comparative environments used by the included studies were different outdoor play environments such as traditional, manufactured and mixed play spaces rather than a true control, such as an indoor environment. Thus, it is difficult to adequately substantiate the findings of this review when the comparator environment was similar to the control. This may represent challenges for future implementation of nature play environments where the evidence base lacks consistency in terms of defining nature play and applying nature play to different contexts.

## Limitations

While this review used a comprehensive and robust methodology, there are still some notable limitations. The resulting evidence base had many concerns regarding the methodological quality of the included studies. The main areas of concern were sampling methods (justification, generalisability and description), lack of reporting around statistical significance (reporting of results), validity and reliability of outcomes measures, dropouts not reported and low-level research designs. Most studies did not explicitly state if the children included in the research had any pre-existing medical conditions. While this may mean all children, who participated in the research were healthy, such ambiguity could be addressed by clearer reporting. Variations in study designs also made it difficult to establish generalisability of the findings due to the study design methodology lacking a robust and rigorous nature. As nature play can have a broad range of impacts on children’s health and development, different studies focussed on different outcomes of interest. While this was outlined in the protocol stage of this review, and the reason as to why outcomes were not included in the search strategy, it was found that the exposure/intervention, outcomes and outcome measures were highly variable. This made it difficult to make a direct comparison of results between studies, meaning that a meta-analysis was not possible and descriptive synthesis was undertaken. While this systematic review process was conducted and reported within the current guidelines for best practice (PRISMA), it may still be subject to publication and language bias, although strategies were undertaken to reduce this impact. For example, to reduce publication bias a grey literature search strategy was implemented as well as secondary searching such as pearling. However, while strategies were in place to combat this bias, the imprecise nature of grey literature guidelines for best practice may have contributed to some level of publication bias. Similarly, due to insufficient access and resource limitations, the search strategy was limited to the English language. However, as a comprehensive search strategy was undertaken which identified that studies were from countries where English was spoken as a second language, language bias was minimised yet, not completely avoided. Another limitation of this review was the inclusion of research from the quantitative research paradigm. While this is appropriate given the nature of the review question with its focus on impact of nature play, this review does not shed light on participants experiences from, and perspectives of, engaging in nature play. This knowledge gap could be addressed through future qualitative meta-synthesis reviews.

## Conclusions

The purpose of this research was to conduct a systematic review examining the impacts of nature play on the health and developmental outcomes of children aged 2–12 years. From the modest body of evidence identified (n = 16) the findings suggest that nature play may positively impact upon aspects of children’s health and development, particularly PA and cognitive development. One of the more significant findings to emerge from this review was in regard to PA outcomes. Physical activity, when comparing nature play to traditional outdoor play spaces, was shown to offer similar PA outcomes. The importance of this finding supports current claims that engaging in outdoor play is more effective in increasing PA in children in both contexts. Notably, these findings also suggest that nature play positively impacts upon children’s cognitive development, particularly in affording imaginative play. These findings together may suggest that the behaviours children engage in outdoors may be an important characteristic when comparing nature play and traditional play spaces. The weight of the findings may be affected by several methodological concerns found within the study designs (lack of a true control), exposure/Intervention consistency, outcome measures used and lack of statistical reporting. Therefore, the recommendations for this review should be considered with caution. Nevertheless, the findings and implications of this review make several contributions to the evidence base on nature play.

## Implications for future research and practice

While the evidence base has identified some support for the positive impact of nature play on children’s health in terms of PA and cognitive development, significant methodological concerns were found in terms of the quality of the evidence. This highlights the need for future research to employ stronger level study designs, such as RCTs, which are considered a high level of evidence against the NHMRC evidence hierarchy. Future research should also address two key issues. Firstly, there is a need for a standardised tool (with established reliability/validity) to observe and characterise children’s play behaviour. The development of a such a tool would ensure consistency when evaluating children’s cognitive/play behaviours, enabling the comparison and pooling of research findings to produce a more robust evidence base for academics, health practitioners, educators and policy/decision-makers. This improved evidence base can then be used to inform educational approaches in training future educators and to develop evidence-informed policy on funding and sustaining nature play spaces. Secondly, there remains ambiguity in the literature around what constitutes a nature play environment—therefore a universal definition should be developed to inform future research. Such research could bring together key stakeholders (educators, consumers, health practitioners, policymakers, play space designers etc) to develop a universally agreed definition of nature play through consensus-based research (such as Delphi, nominal group technique). A universal definition would also afford the development of guidelines to assist policy-makers, educators and practitioners to design, implement and evaluate nature play spaces and experiences for children. Nature play guidelines may also assist in defining what areas are considered nature play in different settings and environmental contexts. The above-mentioned approaches may aid the creation of optimal play spaces and experiences for children to maximise the health and developmental outcomes highlighted in this review.

## Supporting information

S1 Appendix Prisma checklist. (PDF)

S2 Appendix Modified McMaster tool. (PDF)

S3 Appendix Medline search terms and MESH headings. (JPG)

S4 Appendix PROSPERO protocol. (PDF)
