# Supplementary material for: The impacts of unstructured nature play on health in early childhood development: A systematic review
Source: PLoS One. 2020 Feb 13;15(2):e0229006. doi: 10.1371/journal.pone.0229006 (PMC7018039; doi:10.1371/journal.pone.0229006)
Supplement: S2 Appendix — (PDF) [file pone.0229006.s002.pdf]

Nature Play Systematic Review
Kylie Dankiw
Guidelines for Modified McMaster CA tool

McMaster Quantitative Critical Appraisal Tool					
**NO.**	**CRITERION**	**Response**			
**1**	**STUDY PURPOSE**				
	Was the study purpose stated clearly?	Yes	No		
**2**	**LITERATURE**				
	Was the relevant background literature reviewed?	Yes	No		
**3**	**DESIGN**				
	What was the study design?				
**4**	**SAMPLE**				
**4a**	N=				
**4b**	Was the sample described in detail?	Yes	No		
**4c**	Was the sample size justified?	Yes	No		N/A
**5**	**OUTCOMES**				
**5a**	Were the outcome measures reliable?	Yes	No	NAD	
**5b**	Were the outcome measures valid?	Yes	No	NAD	
**6**	**INTERVENTION**				
**6a**	The intervention was described in detail?	Yes	No	NAD	
**6b**	Contamination was avoided?	Yes	No	NAD	N/A
**6c**	Co intervention was avoided?	Yes	No	NAD	N/A
**7**	**RESULTS**				
**7a**	Results were reported in terms of statistical significance?	Yes	No	NAD	N/A
**7b**	Were the analysis method(s) appropriate?	Yes	No	NAD	
**7c**	Clinical importance was reported?	Yes	No	NAD	
**7d**	Drop outs were reported?	Yes	No		
**8**	**CONCLUSIONS AND CLINICAL IMPLICATIONS**				
	Were the conclusions appropriate given the study methods and results?	Yes	No		

Law M, Stewart D, Pollock N, Letts L, Bosch, J & Westmorland, M 1998, *Critical Review Form – Quantitative Studies,* Occupational Therapy Evidence-Based Practice Research Group, McMaster University, Canada

Discussed in meeting on Thursday the 20th December 2018 with Margarita, Katherine and Saravana:

Q4. Sample:

- Studies do not have to specifically state inclusion/exclusion criteria to get a YES. However, they need to have described the sample in terms of characteristics relevant to the population. Such as ethnicity, demographics, age, gender, etc.

Q5. Outcomes:

- In terms of reliability if the authors have used references and cited these when describing an outcomes measure this is acceptable and would get a YES. However, if the author has 3 outcomes measures and has only reported/discussed or cited a reliability reference for 1 out of the 3 this would receive a NO. Yet if they have reported a reliability reference or reported/discussed reliability for the PRIMARY outcome and not for a secondary outcome this would receive a YES.

Q6. Intervention:

- 6b Contamination: only include if there are two groups of people receiving the intervention. Otherwise write NOT APPLICABLE if only one group.

6c Co-intervention: Write NA (not reported) if the authors did not state how they avoided If participants receive another form of influence at the same time as the study exposure, for example if participants attended a nature play educational program before intervention. Or if they reported novelty periods this would be a YES.

Q7. Results:

- 7a results reported as statistically significant:
  - If the study design and aims of the study were to make associations and/or determine the effectiveness of the intervention and reported p-values and confidence intervals, then this would be a YES. However, if it was a descriptive study and reported descriptive statistics then this would also be a YES (as a descriptive study aims to describe a phenomenon therefore would not use p-value or statically significant tests)
- 7b Were analysis methods appropriate?
  - Similarly, to above, if a study which used a study design with an intervention and used descriptive statistics rather than confidence intervals or p-values as their analysis this would be a NO. If a study used a descriptive methodology and used descriptive statistics this would be a YES.
- 7c Clinical importance reported
  - Refers to implications/importance of findings for teachers ECE's, curriculum, schools etc.
- 7d Drop outs reported
  - Many studies have not reported on this, yet if they have stated the initial sample at the beginning and in their analysis the number is the same then this would be a YES. The author(s) do not have to go into detail about why participants dropped out.

Q8 Conclusions:

- If a study used descriptive methodology and reported in the conclusion section that the outcomes were caused by the exposure and made statements that is was because of the exposure (made over reaching statements) then this would be a NO. Because it is descriptive it cannot determine causality of the outcomes so this is a NO.
