# Supplementary figures and images for: The impacts of unstructured nature play on health in early childhood development: A systematic review
Source: PLoS One. 2020 Feb 13;15(2):e0229006. doi: 10.1371/journal.pone.0229006 (PMC7018039; doi:10.1371/journal.pone.0229006)

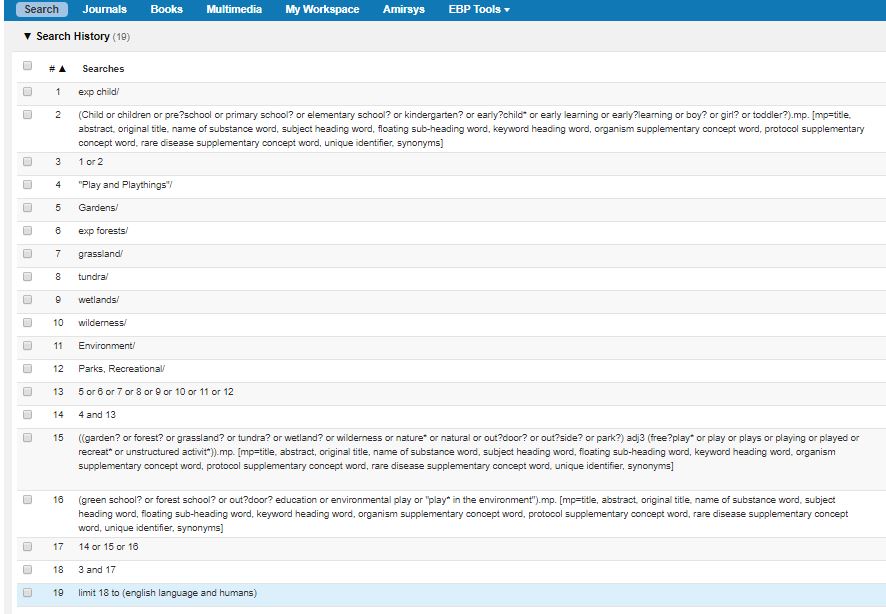

Supplement: S3 Appendix — (JPG) [file pone.0229006.s003.jpg]
