# Supplementary material for: The impacts of unstructured nature play on health in early childhood development: A systematic review
Source: PLoS One. 2020 Feb 13;15(2):e0229006. doi: 10.1371/journal.pone.0229006 (PMC7018039; doi:10.1371/journal.pone.0229006)
Supplement: S4 Appendix — (PDF) [file pone.0229006.s004.pdf]

The associations between 'nature play' and health in early childhood development: a systematic review

Margarita Tsiros, Katherine Baldock, Saravana Kumar, Kylie Dankiw

Citation

Margarita Tsiros, Katherine Baldock, Saravana Kumar, Kylie Dankiw. The associations between 'nature play' and health in early childhood development: a systematic review. PROSPERO 2018 CRD42018084764 Available from:
http://www.crd.york.ac.uk/PROSPERO/display_record.php?ID=CRD42018084764

Review question

What associations can be made between 'nature play' and health and/or developmental outcomes in children aged between 2-12 years of age?

Searches

MEDLINE; ERIC; Embase; PsycINFO; The Cochrane Library; Joanna Briggs Institute; Emcare. The references of relevant articles will be search to identify potential additional articles (pearling). Grey literature searching through an internet web engine (Google and Google Scholar) will be undertaken to identify any additional publications such as, Nature Play SA and South Australian Department for Education and Development. All databases will be searched from data base inception to current. Language (English) restrictions will also be applied.

Types of study to be included

Primary research studies with a particular focus on quantitative research paradigm.

Condition or domain being studied

The purpose of this systematic review is to identify in children between the ages of 2-12 what, if any, associations can be made in between health and development outcomes and participating in nature play activities. Therefore, this systematic review does not have a focus on a singular condition or domain.

Participants/population

Inclusions: Children aged 2-12, female and male, parent and teachers (when used as proxy for capturing outcomes).

Exclusion: Pre-existing physical/mental/neurological health impediments, adults, children above 13, High School children.

Intervention(s), exposure(s)

Inclusion: Nature play, nature-based play, green schools, forest schools, outdoor play, active outdoor play, nature programs, outdoor education.

Exclusion: Indoor sport, outdoor sport, sporting grounds.

Comparator(s)/control

Not applicable.

Context

Main outcome(s)

Including but not limited to:

Social (maturity, cooperation, collaborative involvement, interactions with others, solitary play, group play).

Emotional (stability, stressors, enablers).

Physical (intensity, duration, gross motor skills).

Mental (resilience, happiness, quality of life, self-efficacy).

Intellectual/educational (challenges, knowledge, problem solving skills).

Additional outcome(s)

Barriers and enablers of nature play.

Data extraction (selection and coding)

Title and abstracts identified by the electronic database searches will be screened for potential inclusion by two reviewers. In cases where a decision for exclusion or potential inclusion cannot be made by the title/abstract, the full text will be retrieved. Any disagreements will be discussed and the final inclusion articles will be reviewed by a third reviewer. During this process, software tools such as Endnote and Covindence will be utilised.

Risk of bias (quality) assessment

The McMaster Critical Appraisal Tool – Quantitative Studies will be used to assess the quality of the included studies. This will be undertaken by two independent reviewers and any differences will be addressed through discussion with a third independent reviewer. Studies will not be excluded based on the quality score. However, this information will be used to report, analyse and discuss the overall review findings.

Strategy for data synthesis

Pooling of data by meta-analysis will be undertaken where similar populations, interventions, outcomes and designs are found and the research team determines if the heterogeneity is low. Heterogeneity will be assessed through methodological means as well as subjectively and formally through I^2 statistic. I^2 statistic values of 25%, 50% and 75% represent low, moderate and severe heterogeneity. For the purpose of this review, if the I^2 statistic value was greater than 50%, then readers will be notified of substantial heterogeneity and cautioned regarding interpreting aggregated results.

To determine the impact of nature play compared to the routine play or combined relationship of nature play, the mean difference or standardised mean difference (SMD) and 95% confidence interval (95% CI) will be calculated for each reported outcome using Cochrane Review Manager (V.5). To calculate SMDs the difference in mean scores between the nature play and routine play will be divided by the pooled standard deviation. SMDs will be considered statistically significant if their 95% CI does not cross zero. Interpretation of the strength of the SMD statistics will be based on guidelines suggested by Cohen where a small effect equal or less than 0.2, medium effect is equal or greater than 0.5 and a large effect is equal or greater than 0.8).

If studies are not homogenous, descriptive synthesis will be conducted. Descriptive statistics will be undertaken in Microsoft Excel 2010 (Microsoft Corp, Redmond Washington, USA). Tables will be used to collate all information at one place to use it appropriately for review. The FORM framework will be used after descriptive synthesis is conducted to reduce bias of the researcher. The FORM framework focuses on the descriptive information at the evidence-base of included studies across 5 categories to formulate and grade recommendations for practice guidelines (Hillier et al. 2011).

Analysis of subgroups or subsets

None planned.

Contact details for further information

Kylie Dankiw
danky006@mymail.unisa.edu.au

Organisational affiliation of the review

The University of South Australia
https://www.unisa.edu.au/

Review team members and their organisational affiliations

Dr Margarita Tsiros. The University of South Australia
Dr Katherine Baldock. The University of South Australia
Dr Saravana Kumar. The University of South Australia
Miss Kylie Dankiw. The University of South Australia

Type and method of review
Meta-analysis, Systematic review

Anticipated or actual start date
05 January 2018

Anticipated completion date
05 January 2019

Funding sources/sponsors
No funding

Conflicts of interest

Language
English

Country
Australia

Stage of review
Review Ongoing

Subject index terms status
Subject indexing assigned by CRD

Subject index terms
Child Development; Child, Preschool; Humans; Risk Factors

Date of registration in PROSPERO
16 January 2018

Date of publication of this version
11 July 2018

Details of any existing review of the same topic by the same authors

Stage of review at time of this submission

Stage	Started	Completed
Preliminary searches	Yes	No
Piloting of the study selection process	No	No
Formal screening of search results against eligibility criteria	No	No
Data extraction	No	No
Risk of bias (quality) assessment	No	No
Data analysis	No	No

Versions
16 January 2018

PROSPERO
This information has been provided by the named contact for this review. CRD has accepted this information in good faith and registered the review in PROSPERO. The registrant confirms that the information supplied for this submission is accurate and complete. CRD bears no responsibility or liability for the content of this registration record, any associated files or external websites.
